# Uncharted Territory: The First Case of Cardiac Myxoma in a Patient With NF2

**DOI:** 10.1016/j.jaccas.2025.104155

**Published:** 2025-07-23

**Authors:** Muhammad Salman Sabri, Zinya Talukder, Naila Ijaz, Joseph Neri

**Affiliations:** a Department of Internal Medicine, Jefferson Abington Hospital, Abington, Pennsylvania, USA; b Department of Cardiology, Jefferson Abington Hospital, Abington, Pennsylvania, USA

**Keywords:** cardiac myxoma, meningioma, neurofibromatosis type 2, vestibular schwannoma

## Abstract

**Background:**

Cardiac myxoma is a benign tumor, typically originating in the left atrium, and is often linked to Carney complex. In our literature review, there are no case reports of cardiac myxoma in patients with neurofibromatosis type 2 (NF2).

**Case Summary:**

A 60-year-old man presented with dizziness and was found to have meningioma and vestibular schwannoma, consistent with NF2. Cardiac myxoma was incidentally diagnosed through echocardiography and confirmed by cardiac magnetic resonance imaging and tissue pathology. The mass was surgically resected. The patient was offered genetic testing; however, he decided to not undergo further testing.

**Discussion:**

NF2 is an inherited autosomal dominant disorder associated with vestibular schwannoma, meningioma, ependymoma, cataract, lenticular opacities, and retinal hamartomas.

**Take-Home Message:**

Further research is needed to investigate the potential risk of cardiac myxoma in patients with NF2 and the benefit of routine screening for myxomas in this population.

## Visual Summary

**Figure:**
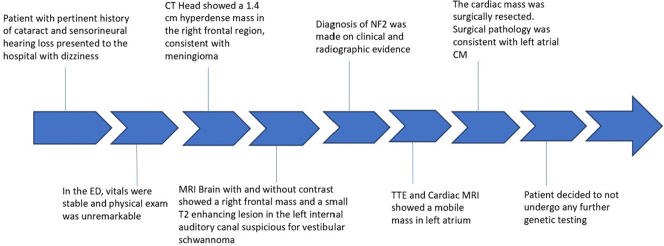

**Visual Summary:**
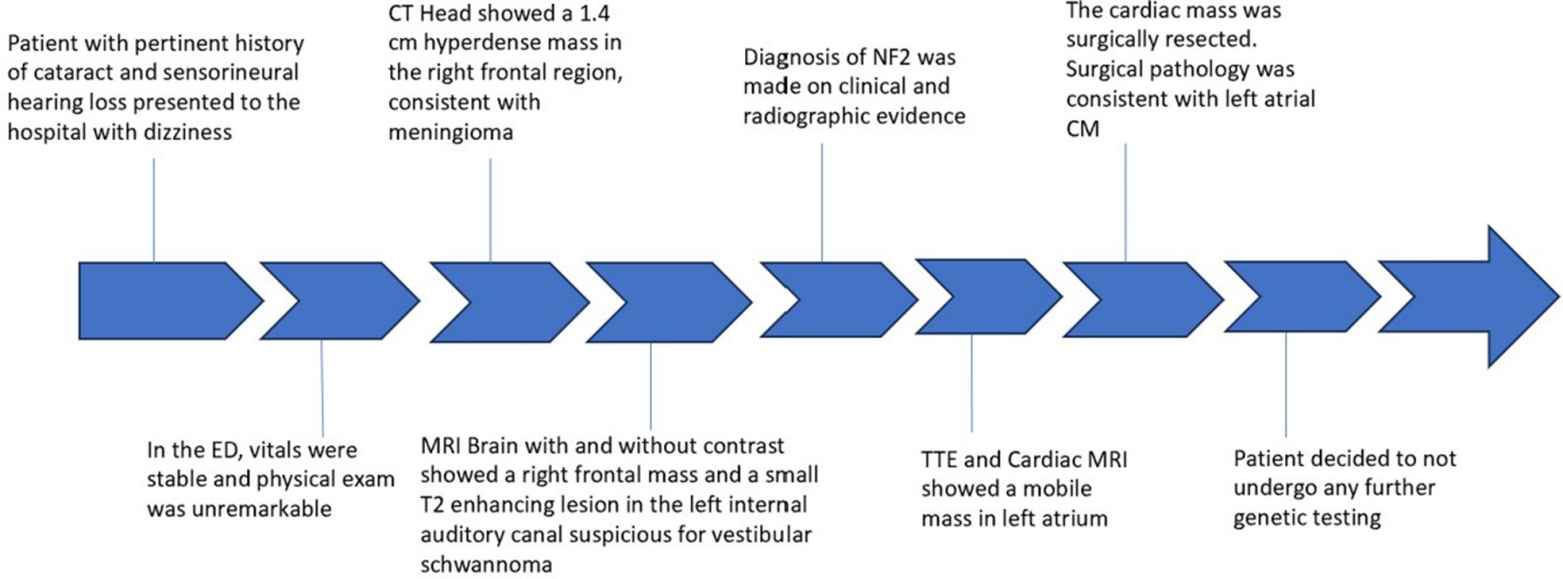
A Rare Intersection: Cardiac Myxoma in NF2

**Take-Home Messages**

• NF2 is linked to meningiomas, vestibular schwannomas, and ophthalmologic findings such as cataracts.
• Although intracranial tumors are more common in patients with NF2, cardiac tumors, particularly myxomas, may also occur.
• Patients with NF2 who present with cardiogenic symptoms, especially syncope, should be evaluated for potential intracardiac tumors, such as cardiac myxoma, as demonstrated in our case.

## History of Presentation

A 60-year-old man presented with dizziness. The patient reported that he began experiencing lightheadedness while cooking at home. He stated that his symptoms progressively escalated to nausea, diaphoresis, and a sensation of impending syncope. He denied family history of genetic disorders, cardiovascular disease, stroke, or brain tumors. On arrival, he was hemodynamically stable, afebrile, and normotensive, with a blood pressure of 131/63 mm Hg and a heart rate of 75 beats/min. The physical examination demonstrated no focal neurologic deficits, chest clear to auscultation, normal heart sounds, and no carotid bruit.

## Past Medical History

The patient's medical history was significant for cataract, hypertension, sensorineural hearing loss, and obstructive sleep apnea.

## Differential Diagnosis

Given the patient's presentation, stable vital signs, and unremarkable physical examination, our differential diagnosis included vasovagal syncope, labyrinthitis, vestibular neuritis, and/or cardiac arrhythmia.

## Investigations

**Figure 1:**
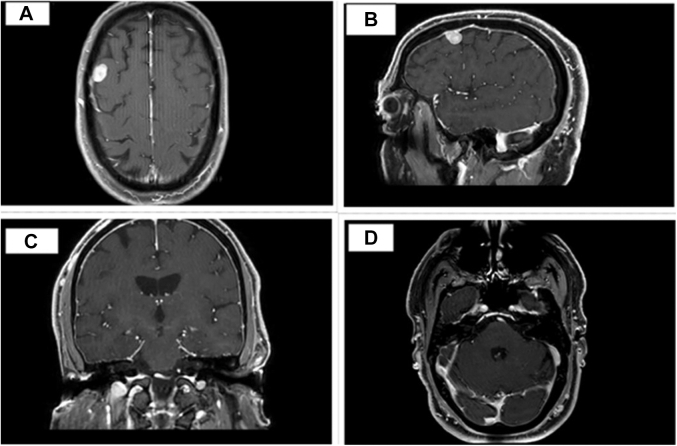
Magnetic Resonance Imaging Magnetic resonance imaging (MRI) of the brain shows a right frontal lesion (A, B), consistent with meningioma. Furthermore, a small enhancing lesion in the left internal auditory canal (C, D) is consistent with vestibular schwannoma.

**Figure 2:**
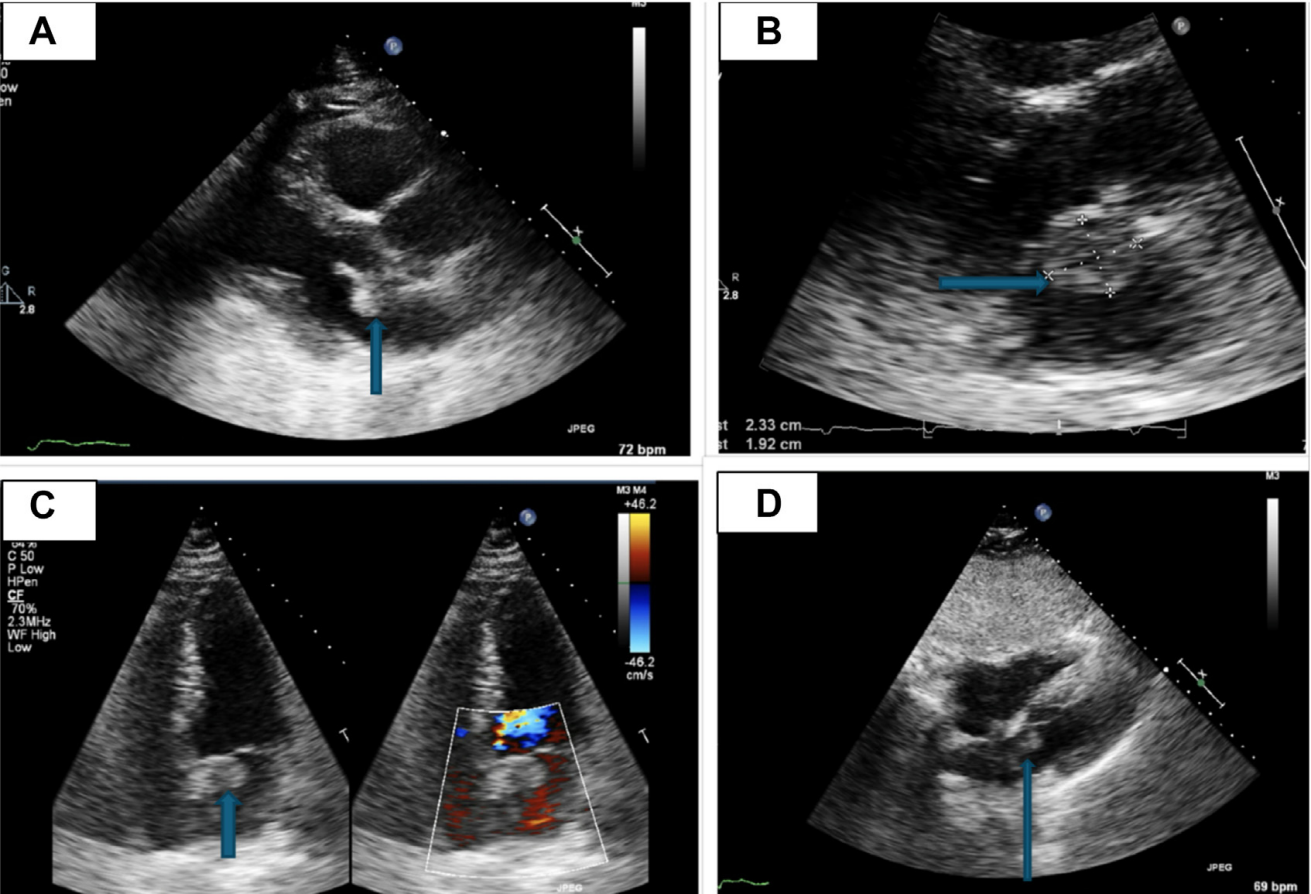
Transthoracic Echocardiogram Transthoracic echocardiogram (TTE) reveals a 2.3 × 1.9 cm mobile mass in the left atrium (A, B), indicated by blue arrows. The mass appears attached to the interatrial septum, as shown by arrows in C and D, consistent with an atrial myxoma.

**Figure 3:**
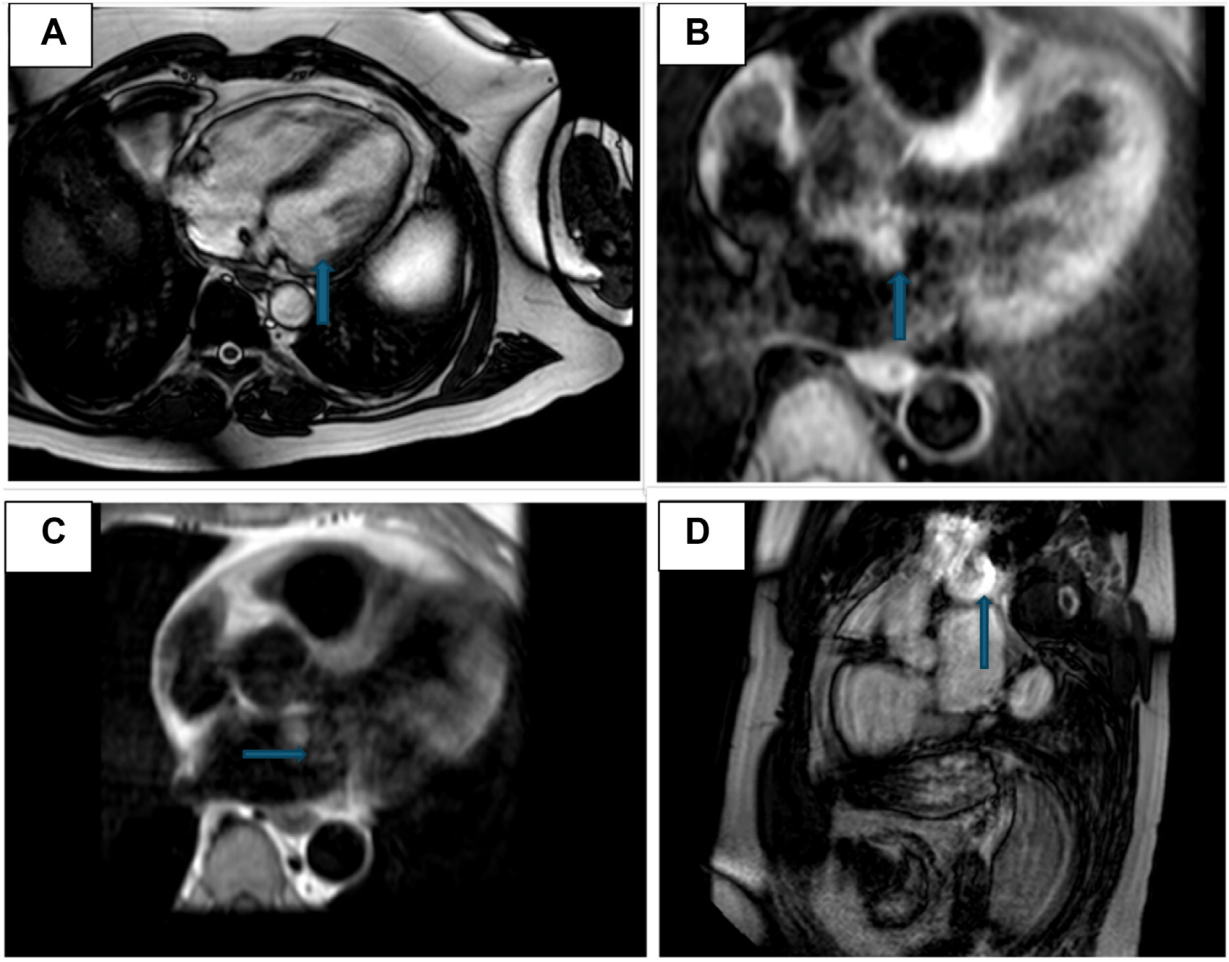
Cardiac Magnetic Resonance Imaging Cardiac magnetic resonance imaging (MRI) shows a 15 × 14 mm mass in the left atrium along the anteromedial wall (A), indicated by an arrow. The lesion appears isointense to skeletal muscle on T1-weighted images (B, C) and hyperintense on the T2-weighted sequence (D), as highlighted by arrows. Its location adjacent to the aortic root and imaging characteristics are highly suggestive of a myxoma.

**Figure 4:**
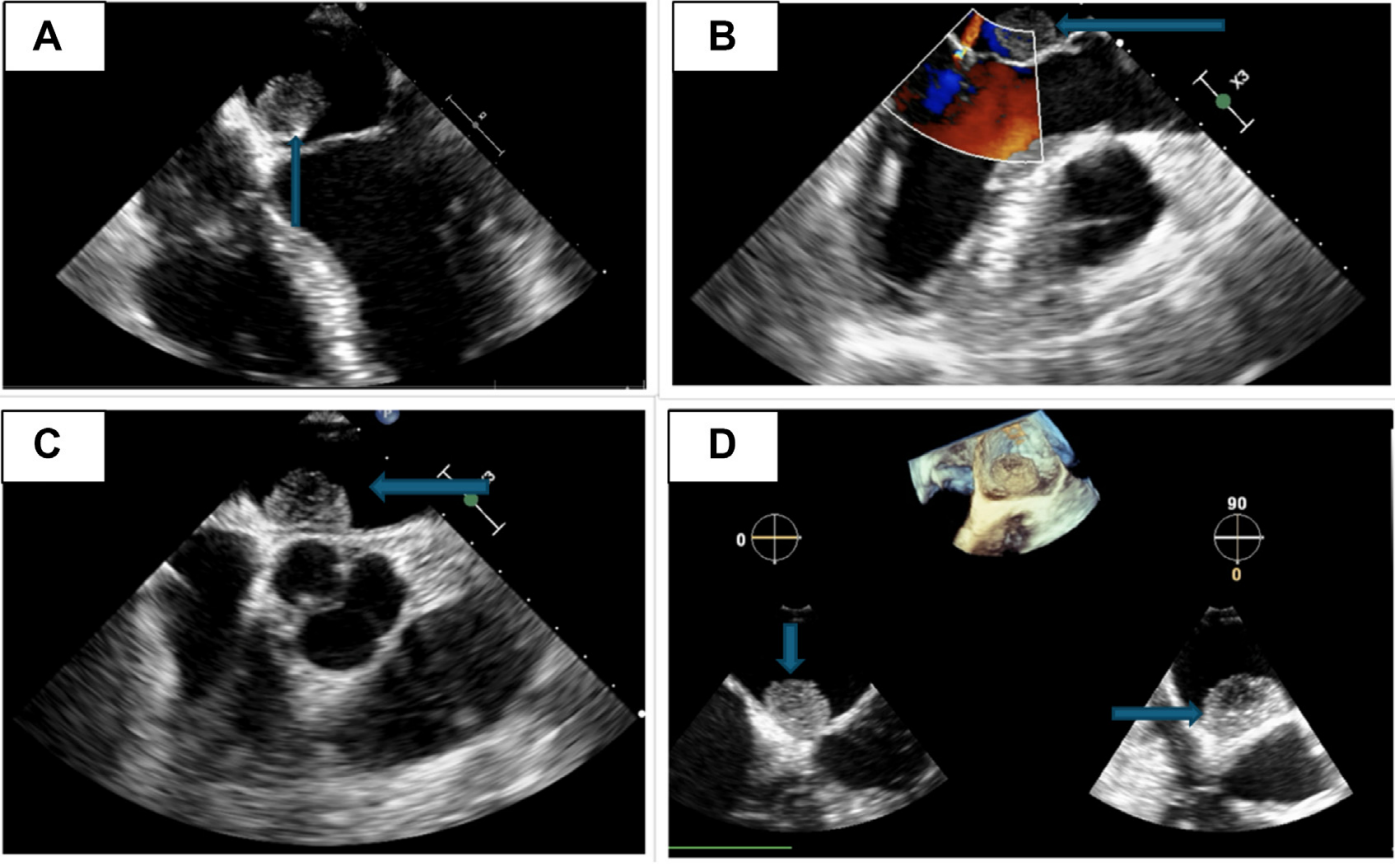
Transesophageal Echocardiogram Transesophageal echocardiogram reveals a 2.3 × 1.7 cm mass in the left atrium (A), indicated by an arrow, consistent with a myxoma. The mass is attached to the interatrial septum (B) and is positioned anteriorly near the aortic valve (C), as shown by arrows. It appears broad-based without a distinct stalk and is located approximately 1 cm above the mitral annulus (D), with no evidence of obstruction to mitral inflow.

Laboratory work-up was unremarkable. An electrocardiogram revealed normal sinus rhythm with a left anterior fascicular block. A computed tomography scan of the head showed a 1.4-cm hyperdense mass in the right frontal region, consistent with meningioma. Magnetic resonance imaging (MRI) of the brain (Figure 1) confirmed multiple acute infarcts in the bilateral cerebellar hemispheres and a right frontal mass, consistent with meningioma. Additionally, a small T2-enhancing lesion in the left internal auditory canal raised suspicion for a vestibular schwannoma. Diagnosis of neurofibromatosis type 2 (NF2) was made based on clinical and radiographic findings. A transthoracic echocardiogram (TTE) (Figure 2) revealed a 2.3 × 1.9-cm mobile mass in the left atrium, consistent with cardiac myxoma. Cardiac catheterization excluded coronary artery disease. Cardiac MRI (Figure 3) identified a 15 × 14 mm mass in the left atrium along the anteromedial wall, which appeared isointense to skeletal muscle on the T1 sequence and hyperintense on the T2 sequence. The mass was located adjacent to the aortic root and was most likely a myxoma. The left ventricular ejection fraction was 53%, with no signs of delayed myocardial enhancement, effectively excluding scar tissue, infarction, or infiltrative disease. Intraoperative transesophageal echocardiography (Figure 4) identified a 2.3 × 1.7 cm left atrial mass, consistent with a cardiac myxoma, located along the interatrial septum and positioned more anteriorly near the aortic valve. The mass was broad based, without a distinct stalk, and situated approximately 1 cm above the mitral annulus, with no evidence of mitral inflow obstruction.

## Management

The mass was surgically resected through a sternotomy with cardiopulmonary bypass. Pathologic examination of the surgical specimen revealed multiple fragments of red-white gelatinous tissue with a villous appearance, consistent with a left atrial cardiac myxoma. Genetic testing was offered; however, the patient decided to not undergo further testing.

## Discussion

The reported prevalence of cardiac myxoma, a benign primary tumor of the heart, is 0.03% in the general population. It originates primarily from the left atrium and is either sporadic in nature or associated with Carney complex. Our literature review found no case report or documentation of cardiac myxoma in a patient with NF2.

NF2 is an autosomal dominant disorder that results from loss-of-function mutations in the *NF2* gene located on chromosome 22q11.2.^1^ This gene encodes Merlin protein, which regulates cell growth, especially in Schwann cells. Merlin protein deficiency results in increased tyrosine kinase level, which leads to cell growth. More than 50% of patients with NF2 have new mutations. Additionally, 20% to 30% of patients without a family history exhibit mosaicism, which can lead to mild generalized or unilateral disease.^2^ Because our patient did not have a family history of genetic disorders, it is likely that he had a new mutation resulting in NF2.

NF2 is diagnosed based on clinical criteria and/or genetic testing. Specifically, patients should meet one of the following criteria: bilateral vestibular schwannomas, an identical NF2 pathogenic variant in 2 or more anatomically distinct NF2-related tumors, 2 major criteria, or 1 major and 2 minor criteria.^1^ Major criteria include unilateral vestibular schwannoma, a first-degree relative other than a sibling with NF2, 2 or more meningiomas, and NF2 pathogenic variant in an unaffected tissue. Minor criteria include ependymoma, nonvestibular schwannoma, a single meningioma, cataract, and retinal hamartoma.^3^ Our patient had a constellation of findings consistent with a diagnosis of NF2—MRI findings of a right frontal mass consistent with meningioma, a small lesion in the left internal auditory canal suspicious for a vestibular schwannoma, and cataract—thus meeting 1 major and 2 minor criteria. Genetic testing is recommended for patients with suspected NF2, although it is not mandatory for diagnosis when sufficient clinical and radiographic evidence is available.^4^ Nevertheless, genetic testing can be valuable in assessing severity of the disease and determining whether family members should undergo screening for NF2.^4^

Most tumors related to NF2 are not cancerous; however, patients with NF2 undergo routine monitoring as these tumors can grow to affect local structures and cause distressing symptoms. Children of affected patients are considered to have a 50% risk of NF2, and screening for NF2 can start at birth. Patients with NF2 undergo periodic MRI scans for vestibular schwannoma and spinal surveillance starting at age 10 with varying frequencies based on age and tumor presence.^5^ Given that cardiac involvement is rare in NF2, cardiac imaging is not routinely performed in patients with NF2. However, if left undiagnosed, cardiac myxomas can have detrimental effects from their obstructive and embolic features.

Patients with NF2 can present with symptoms of hearing loss, problems with balance, or seizures. Our patient had sensorineural hearing loss; however, he did not undergo an appropriate work-up, which may have led to an earlier diagnosis of NF2. There is rarely any cardiovascular involvement in patients with NF2. Patients with segmental neurofibromatosis can have hypertrophic cardiomyopathy, hypertension, and coronary artery aneurysm; however, this has been reported with neurofibromatosis type 1, not NF2.^6^ Cases of cardiac neurofibroma have rarely been associated with neurofibromatosis type 1; however, they have never been documented in patients with NF2. Therefore, the presence of a cardiac myxoma in this patient with NF2 presents a unique case.

Cardiac myxomas are considered common primary neoplasms of the heart. A cardiac myxoma most commonly occurs in middle-aged persons and has a female-to-male occurrence ratio of 3:1. Most commonly, they are sporadic in nature but can also be familial as part of Carney complex. The 2 morphologic forms are polypoid, which manifests with obstructive features, and papillary, which is prone to embolization.^7^ The multiple acute infarcts in the bilateral cerebellar hemispheres visualized in our patient's brain MRI scan suggest embolization from his cardiac myxoma. The clinical manifestation of cardiac myxoma varies widely, ranging from silent asymptomatic cases, when the tumor is <4 cm, to unexpected sudden death due to flow obstruction or embolization.^8^^,^^9^ Tumor emboli can travel to any vascular bed and can remain viable at the site of embolization resulting in formation of distant metastatic tumors. As a result, although cardiac myxomas are commonly benign tumors, they can have detrimental effects from local invasion, recurrence after resection, and distant metastasis.^10^ Data suggest a recurrence rate of 3% in sporadic cases and 20% in Carney complex.^7^

TTE is the diagnostic study of choice. An echogenic mobile mass within the atrium attached to the interatrial septum through a stalk are the telltale features of cardiac myxoma on echocardiogram. TTE in our patient showed a mobile mass attached to the interatrial septum in the left atrium. Intraoperative transesophageal echocardiography confirmed the location of the mass and further described it as broad based, without a distinct stalk, and situated approximately 1 cm above the mitral annulus without any mitral inflow obstruction. Surgical excision is the mainstay of treatment with low operative mortality.^7^

In our patient, a cardiac myxoma was identified in the context of an NF2 diagnosis. Patients with Carney complex may present with cardiac myxomas and schwannomas; however, the schwannomas typically manifest as psammomatous melanotic schwannomas, which do not show enhancement on T2 imaging owing to their melanin content. In contrast, vestibular schwannomas associated with NF2 exhibit T2 enhancement on MRI, as seen in our patient. Additionally, meningiomas are more commonly observed in NF2 than in Carney complex. Our patient also did not exhibit any cutaneous manifestations or endocrinopathy, which made Carney complex less likely, as the patient did not fulfill the diagnostic criteria.^11^ Although cardiac myxoma could be a sporadic event, it may also be related to NF2, and further research is needed to better understand this potential correlation.

## Conclusions

Patients with NF2 are known to be at increased risk for multiple central nervous system tumors, including vestibular schwannomas, meningiomas, and intramedullary tumors (eg, ependymomas), which is why they undergo routine MRI screening of the brain and spine. Cardiac involvement in NF2 is rare, with no reports to our knowledge in the literature. Our case of a patient with NF2 diagnosed with a left atrial cardiac myxoma highlights the need for further investigation into the potential link between NF2 and cardiac myxoma.

## Funding Support and Author Disclosures

The authors have reported that they have no relationships relevant to the contents of this paper to disclose.

## References

[1] Plotkin S.R., Messiaen L., Legius E. (2022). Updated diagnostic criteria and nomenclature for neurofibromatosis type 2 and schwannomatosis: an international consensus recommendation. Genet Med.

[2] Wang M.X., Dillman J.R., Jeffrey G. (2022). Neurofibromatosis from head to toe: what the radiologist needs to know. Radiographics.

[3] Evans D.G., Adam M.P., Feldman J., Mirzaa G.M. (2023). GeneReviews®.

[4] Asthagiri A.R., Parry D.M., Butman J.A. (2009). Neurofibromatosis type 2. Lancet.

[5] Evans D.G. (2009). Neurofibromatosis type 2 (NF2): a clinical and molecular review. Orphanet J Rare Dis.

[6] İncecik F., Hergüner Ö.M., Alınç Erdem S., Altunbaşak Ş. (2015). Neurofibromatosis type 1 and cardiac manifestations. Turk Kardiyol Dern Ars.

[7] Islam A.K.M.M. (2022). Cardiac myxomas: a narrative review. World J Cardiol.

[8] Amano J., Kono T., Wada Y. (2003). Cardiac myxoma: its origin and tumor characteristics. Ann Thorac Cardiovasc Surg.

[9] Sabri M., Qamar S., Ijaz N. (2024). Embolic stroke in a patient with left atrial myxoma. JACC.

[10] Aiello V.D., de Campos F.P. (2016). Cardiac myxoma. Autops Case Rep.

[11] Saleh Y., Hammad B., Almaghraby A. (2018). Carney complex: a rare case of multicentric cardiac myxoma associated with endocrinopathy. Case Rep Cardiol.

